# Effectiveness of interventions to improve employment for people released from prison: systematic review and meta-analysis

**DOI:** 10.1186/s40352-023-00217-w

**Published:** 2023-03-14

**Authors:** Catriona Connell, Mary Birken, Hannah Carver, Tamara Brown, Jessica Greenhalgh

**Affiliations:** 1 Salvation Army Centre for Addiction Services and Research, University of Stirling, Stirling, UK; 2 Division of Psychiatry, University College London, London, UK; 3 Leeds Beckett University, Leeds, UK; 4 Drugs Research Network Scotland, University of Stirling, Stirling, UK

**Keywords:** Offenders, Employment, Meta-analysis, Systematic review, Rehabilitation, Reintegration, Resettlement

## Abstract

**Background:**

People released from prison experience complex health challenges in addition to challenges resettling into the community. Consequently, employment rates are low. Participating in good quality employment can support good health and is protective against future reoffending. Multiple interventions are provided to support people into employment on release. The effectiveness of interventions for improving employment outcomes has not previously been evaluated in a meta-analysis.

**Aim:**

Our objective was to examine the effectiveness of interventions to improve employment following release from prison.

**Method:**

We searched seven databases and three trial registries for peer reviewed randomised controlled trials (RCTs), published since 2010, that included adults and measured an employment outcome(s). We conducted meta-analysis using random effects models with sub-group and sensitivity analyses. We appraised bias risk per outcome, and incorporated this into an assessment of the certainty estimates for each outcome. A group of people with experience of imprisonment met with us throughout the project to inform our search strategy and interpretation of results.

**Results:**

We included 12 RCTs (2,875 participants) which were all conducted in the USA. Few outcomes were of low risk of bias. Intervention participants were 2.5 times more likely to work at least one day (95% CI:1.82–3.43) and worked more days over 12 months (MD = 59.07, 95% CI:15.83–102.32) compared to controls. There was no effect on average employment status or employment at study end. There is moderate certainty in these estimates.

**Conclusion:**

Interventions can improve some employment outcomes for people released from prison. More evidence is required to establish effective interventions for sustaining quality employment, particularly outside the USA, and which consider outcomes for different groups of people released, such as women or those with health or substance use needs.

**Supplementary Information:**

The online version contains supplementary material available at 10.1186/s40352-023-00217-w.

## Introduction

### Why employment following imprisonment matters

The global prison population is estimated to exceed 10.77 million people (Fair & Walmsley, 2021). The prison population experience disproportionately high rates of health conditions and substance use disorders, and often experience social and economic deprivation prior to imprisonment (Enggist et al., 2014). Almost all people in prison will be released to the community. Given the multiple complex needs of those released (Enggist et al., 2014), disruption to social structures when someone is removed from the community resettlement in areas that may be socially and economically disadvantaged (Morenoff & Harding, 2014), and the stigma and discrimination experienced from employers, it is unsurprising that people released from prison have ongoing poor health and employment rates markedly below those of the general population (Brunton-Smith & Hopkins, 2014; Couloute & Kopf, 2018; Cutcher et al., 2014). Enabling those released from prison to successfully (re)join the labour market is an important ambition due to the association between unemployment and poor health (McKee-Ryan et al., 2005) and the association between employment and avoiding reoffending (Olver et al., 2014), which both have important implications for the individual, the communities they return to and society as a whole (Morenoff & Harding, 2014).

#### Employment and health

Unemployment is consistently demonstrated to be associated with poor health, particularly mental health (McKee-Ryan et al., 2005). Some groups experience a heightened detrimental effect, such as manual workers and those who are unemployed due to health reasons (Norström et al., 2014). However, whilst unemployment is closely associated with poor health, the relationship between employment and good health is not clear cut. Employment can be a source of stress and contributor to illness, particularly when considering the rise in low quality work opportunities such as insecure work in the ‘gig economy’, low pay that does not enable people to meet their basic financial commitments, and poor working conditions (The Health Foundation, 2020a). Moving into poor quality work can be more harmful to health than remaining unemployed (Chandola & Zhang, 2018). Given the low levels of literacy, wide social disadvantage and frequent resettlement in deprived communities, the opportunities for good quality employment following imprisonment may be limited and thus employment may not equate to health improvements. It is therefore vital that attempts to engage people in employment following imprisonment consider the quality of work that people are encouraged towards.

At community level, there is a strong correlation between employment rates and healthy life expectancy (The Health Foundation, 2020b). People released from prison are often released to areas of social disadvantage, where opportunities for employment and cultures that value employment may both be lower. Remaining unemployed then adds to a community’s overall strain from levels of unemployment (Morenoff & Harding, 2014).

#### Employment and reoffending

Employment is associated with a reduced risk of reoffending (Olver et al., 2014) with multiple theories for how this occurs. Employment may increase ties to ‘conventional society’, providing a prosocial network and informal social controls that discourage offending (Sampson & Laub, 1993). Employment is argued to provide an avenue for developing an identity that is incompatible with criminal activity and which becomes a valuable status to lose (Maruna, 2001). There are also rational choice arguments, that employment provides financial security sufficient to negate the need to choose to make money illegally (Becker, 1968). As with health, it is good quality work that is implicated in avoiding reoffending (Ramakers et al., 2017). Additionally, changes in offending patterns may precede employment changes, suggesting other factors could influence changes in offending (Lee, 2019). Thus, in supporting people released from prison into employment, it is important to consider the quality of work and the wider context of their lives if avoiding reoffending is to be achieved. Benefits of avoiding reoffending may be experienced by the individual through an avoidance of further punishing sanctions, but there are also benefits victims and communities who would be adversely affected by crime.

### Effectiveness of interventions to increase employment following imprisonment

The relationship between employment, health and reoffending (but particularly reoffending) underpins international expectations that prison will provide individual level support to increase employment rates among people released from prison (Council of Europe Publishing, 2006). To a lesser extent, there are interventions seeking to reduce structural barriers in the employment market. For example, ‘Ban the Box’ is a campaign to remove the need to disclose convictions early in job applications, which is intended to increase someone’s chances of securing an interview (Unlock, 2022).

Prisons often offer educational and skill development programmes, work programmes, and supervised work release. These operate on the assumption that addressing the limited education and work experience in the prison population will increase a person’s likelihood of successfully securing future employment. Ellison et al. (2017) conducted a rapid evidence assessment and meta-analysis of five prison-based educational interventions on post-release employment. Whist their analysis suggests 24% increased odds of employment for those in receipt of interventions, this result must be considered of very low certainty as individual study results varied markedly, two of five showed prison education interventions had a negative impact on employment prospects and the authors included non-randomised designs. Nur and Nguyen (2022) conducted a meta-analysis of prison based work and vocational programmes in the USA, including non-randomised designs. When combining all employment outcome types in 11 studies, the overall effect size estimate using log odds was -0.335 (*p* < 0.01). However, their inclusion studies from the USA only, and from over 30 years ago limits transferability of these findings.

Following release, there is range of support for employment provided by statutory and non-statutory services that vary in their approach from focusing singularly on employment, to those which offer more holistic interventions. Additionally, there is variation between programmes which follow a traditional ‘train then place’ approach (where people are anticipated to gradually progress from learning skills, to supported or voluntary work, and ultimately to competitive employment), and those which will seek to place someone directly in work. The latter approach is exemplified by Individual Placement and Support (IPS; Rinaldi et al., 2008). IPS was developed for people with severe mental illnesses and focuses on rapid job search and placement, underpinned by an assumption that once in employment someone will develop the work skills to sustain that job. In a meta-analysis, IPS increased the likelihood of participants with mental illnesses starting employment by 2.4 times when compared to traditional vocational rehabilitation (Modini et al., 2016). The potential of this approach has led to calls for greater evaluation of its effectiveness with justice-involved people (Durcan et al., 2018). The most recent meta-analysis to examine the effectiveness of community-based employment interventions for justice-involved people (not delivered in custody) concluded that interventions are ineffective for reducing reoffending but did not draw a conclusion about their effectiveness in improving employment (Visher et al., 2005). Included studies were conducted in the USA from the 1970s onwards, when labour market, employment practices, legislative and policy environments, and common employment opportunities were markedly different from contemporary USA and international environments.

There is currently limited evidence for the effectiveness of interventions to improve *employment* for people who have been in prison, whether these are delivered in custody or in the community. Our aim was to address this gap in the literature to better inform policymakers and practitioners when commissioning and implementing interventions in a contemporary context. We present a systematic review and meta-analysis of the effectiveness of interventions tested in randomised controlled trials (RCTs) to improve employment for people released from prison. Our primary outcome is employment itself.

## Methods

We conducted a systematic review and meta-analysis of results from RCTs that tested an intervention for its effect on employment among adults released from prison. We pre-registered our protocol on the PROSPERO website (CRD42021274409). Our protocol describes the inclusion of papers that report any social outcome (employment as well as other socially valued activities and roles) and intention to do meta-analysis where feasible. It was only feasible to conduct meta-analysis using studies that reported employment, which we report here using the Preferred Reporting Items for Systematic Reviews and Meta-Analyses (PRISMA) 2020 (Page et al., 2021). 

### Lived experience involvement

We held three meetings with people with experience of post-imprisonment community living to ensure the research had relevance to those it aims to serve, was thoughtfully conducted and communicated, and incorporated experiential knowledge of those with direct relevant experience. Meeting one informed the search strategy. Meetings two and three focused on interpreting the results and considering their implications from the perspective of intervention recipients, which informed our discussion.

### Eligibility criteria

We included RCTs involving adult (18 + years) participants who had been in prison, and that measured employment after release. RCTs could use any comparator and any follow up period. In studies with mixed samples, we only included studies that identified that at least 50% of participants had been in prison. No limits were placed on geography, offence type, prison/sentence type or diagnosis. We included studies published since 2010 to ensure relevance to a contemporary context.

### Information sources

Two authors (CC and MB) searched seven databases selected to cover health, social and criminological literature (ASSIA, CINAHL via EBSCOhost, Cochrane Central Register of Controlled Trials, Criminal Justice Abstracts via EBSCOhost, EMBASE, PsycINFO via EBSCOhost, Web of Science All Databases) in August 2021. We additionally searched trial databases (Clinical trials.gov, ISRCTN and OpenTrials) and conducted forward and backward citation tracking on included studies. We conducted the search in English and screened papers with English language abstracts. We did not exclude studies in other languages if the abstract was in English, determining eligibility using freely available translation software and by contacting authors for clarity and to seek an English language manuscript.

### Search strategy

We combined terms for the population (e.g., offender, criminal), outcome (e.g., work, employment, vocation), community setting (e.g., parole, probation) and study type (randomised controlled trials). We searched each database using subject headings that mapped to our concepts, and free text searches at the title and abstract level, limiting our results to those published from 2010 onwards. Our template and a full copy of our search of EMBASE are presented in Additional File 1.

### Selection process

Two authors (CC, MB) double screened the first 10% of records, achieving agreement on all but one. A third author (HC) made the final decision and CC screened the remainder of the papers in line with the standards agreed. The same two authors independently screened 50% of full text citations. We used Rayyan, an web based platform for facilitating multi-institutional review teams (Ouzzani et al., 2016), to facilitate both stages.

### Data collection process and data items

Three authors (CC, MB, JG) piloted a data extraction tool (available from corresponding author) with one paper and met to determine consistency and relevance of the data extracted. No amendments were required, and these authors independently extracted data from the remaining studies including: participant demographics; details about the setting, intervention, and comparator; outcomes and outcome measures; and results at all reported time points. Where necessary data were not presented in a paper, we contacted study authors by email with responses and additional data sent in most instances.

### Risk of bias assessment

We used the Risk of Bias 2 (RoB2; Sterne et al., 2019) and the cluster RCT extension. RoB2 is a structured assessment with clear rating criteria that facilitates assessment of bias per outcome, rather than a study overall. For example, where a study measured employment in multiple ways (e.g., if work was started and number of days in work), each outcome can be separately appraised for bias risk. The ROB2 attends to five domains: the randomization process (three items), deviations from the intended intervention including awareness of assignment and levels of adherence (seven items), the extent to which outcome data is missing (four outcomes), how outcome data was measured (five outcomes), and whether results are reported in full or selectively (three items). Each domain is rated as low risk, some concerns, or high risk by algorithm (which can be overridden by the assessor) before an overall judgement of bias risk is determined. Some items require a pre-published protocol to identify discrepancies, for example where the outcomes reported have been selected post-hoc for the favourable results they show. Three reviewers worked independently to appraise risk of bias (CC, MB, JG) with one reviewer checking all assessments (CC). We contacted authors for further information where no protocol was identified, to seek clarifications and request further data, which elicited further data in some cases. Results are summarized in Additional File 2.

### Summary measures

For studies reporting dichotomous outcomes (e.g., in employment yes/no, worked at least one day yes/no) we calculated odds ratios. To compare our results with other published estimates we also calculated risk ratios. For studies with continuous outcomes (e.g., no of days worked) we calculated pooled mean differences. Where studies reported time in work but used different measurement units (hours, days, weeks, months) over different time periods, we calculated standardized mean differences. For studies with multiple time points, we took results from the time point closest to that used in other studies in the comparison. Although all outcomes pertained to employment, achieving employment and the amount of time worked are different constructs and thus transforming all results into a single estimate of effect could not be justified theoretically.

### Synthesis methods

We created a summary table to indicate the intervention and control conditions, follow up duration, outcomes, and results for each study. This enabled us to identify where we had three or more studies reporting the same or sufficiently similar outcomes to permit a meaningful meta-analysis.

We conducted meta-analysis in Revman5 (Cochrane Collaboration, 2020), a software package designed to facilitate meta-analyses. We used random effects models for all comparisons due to the variation in interventions and populations. Heterogeneity was assessed using the x^2^ statistic, but due to the relatively small number of studies and differences across populations and interventions per comparison, we also used the I^2^ statistic (reporting the percentage of variability in the effect estimate due to heterogeneity). We followed assumptions that < 40% indicates heterogeneity that may not be important, whilst > 75% indicates considerable heterogeneity (Deeks et al., 2019). To explore heterogeneity, we conducted subgroup analyses. We explored differences between the general prison/community justice population and specific subgroups (e.g., those utilising mental health services). We explored intervention differences based on the setting where the intervention was delivered (prison, community, or both). Where we had several studies of the same intervention (i.e., IPS) and studies of other interventions in the same comparison, we obtained effect estimates for IPS alone. We conducted sensitivity analyses to test the impact on the results when excluding studies of high risk of bias, and when excluding those using only self-report measures of employment.

### Reporting bias assessment

We intended to assess potential for publication bias using funnel plots, which requires 10 or more studies to be sufficiently powered. However, we had too few studies in each comparison to facilitate this.

### Certainty assessment

We used the GRADE approach (Schünemann et al., 2013) to rate the certainty of the evidence about whether employment interventions should be provided for people released from prison and to identify where further research is needed to make clear recommendations. The GRADE approach involves an assessment of each meta-analytic comparison that considers risk of bias, inconsistency, indirectness, and imprecision. Risk of bias draws together the bias risk of the studies included in the comparison, inconsistency arises where there are wide differences between study results, indirectness is where treatments are only comparable by reference to a third treatment or a surrogate outcome/treatment/population is used, and imprecision refers to the level of uncertainty around an estimate and whether confidence intervals around the effect cross a threshold for clinically meaningful change. Results are summarised in Additional File 3.

## Results

### Study selection

**Fig. 1:**
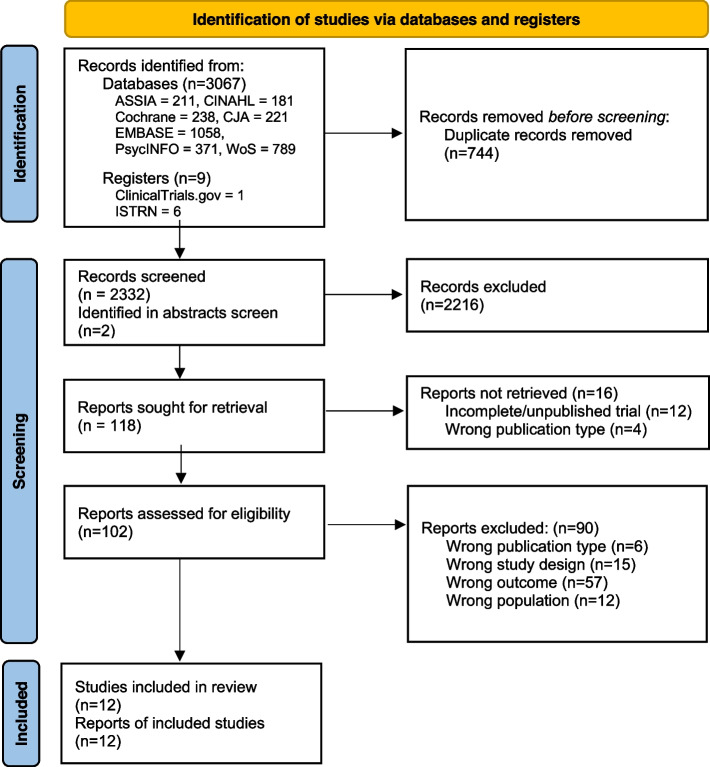
PRISMA Flow Diagram

Following duplicate removal, we screened 2332 records at title and abstract level against our eligibility criteria. 118 were reviewed at full text, resulting in the inclusion of 12 RCTs involving 2,875 participants which reported at least one employment outcome (see Fig. 1). This included one cluster RCT which could not be incorporated into meta-analysis (Polcin et al., 2018).

### Study characteristics

Individual study characteristics are summarized in Table 1 and outlined briefly here.

**Table 1:** Individual study characteristics and results

Study	Population	Intervention	Comparison	n	Follow up (months)	Outcomes	Results
Bond et al. (2015)	Mental illness 77–82% men 48–70% African American 76% prison history Mean age 42.9–44.6	Individual placement and support (IPS) Community based Employment focused Brief vocational assessment followed by rapid individualised job search. Individualised ‘job development’ and accompaniment to interview by employment specialist if desired	Work Choice (Job Club)	87	12	Achieved any employment	31% intervention vs 7% control (N = 85, x^2^ = 7.99, df = 1, *p* < 0.01)
						Days employed	40.5699.2 intervention vs 15.9665.7 control (*N* = 85, MWU = 2.67, *p* < 0.01)
Cook et al. (2015)	General population 100% men 84–86.2% Black 100% prison history Mean age 28	Milwaukee Safe Streets Prisoner Reintegration Initiative (accelerated service access) Transitional Employment included within treatment/support services Accelerated access to pre-release planning, supported employment, social work assessment and support, vocational skills assessment, ‘cognitive reality curriculum’	Usual services	236	12	Achieved any employment	Intervention 42% vs control 39%
						Employment status at 12 months	Intervention 81% vs control 59%
Duwe (2012)	General population 84–93% men 68.6–73.4% Racial/ethnic minority 100% prison history Mean age 36.2–36.9	Minnesota Comprehensive Offender Re-entry Plan (MCORP) Transitional Employment included within treatment/support services Pre-release: Institutional case workers establish a transition accountability plan Post release: MCORP agents help to access services for employment, vocational training, education, housing, chemical health, mentoring, faith-based programming, and income support Combines risk assessment with elements of motivational interviewing, SMART goals, and assistance to access support	Usual services	269	6	Achieved any employment	55% intervention vs 39.2% control (*N* = 249, x^2^ = 5.56, *p* < 0.05)
Farabee et al.. (2014)	General population 78.3–87.3% men 34.3–34.8% Hispanic 19.7–24.3% African American 100% prison history Mean age 35–35.8	Employment-focused re-entry program Community based Employment focused 4 weeks training 40 h per week on job readiness, followed by assistance to find work with access to computers	List of resources and meal voucher	217	12	Ave. employment status last 1 month	Full time employment 26.2% intervention vs 22.0% control (ns) Part time employment 12.6% intervention vs 12.2% control (χ^2^ = .506; df = 2; *p* < .776)
						Ave. employment status last 12 months	Full time employment 29.8% for intervention vs 27.1% control Part-time employment 12.5% for intervention vs 9.4% control group (χ^2^ = .789; df = 2; *p* < .674) (ns)
Fogel et al. (2015)	General population 100% women 54.0–61.7% white 100% prison history Mean age 33.4–34.2	Sexual health intervention Prison based Sexuallt transmitted infection (STI) behavioural intervention with no employment focus 8 × 1.5 h (adapted from an existing evidence-based HIV-STI prevention intervention for ethnic minority women diagnosed with STIs in public health clinics	Usual services	521	6	Employment status at 3 months	Higher proportion of intervention group *unemployed* (77.8% vs 73.1%). Adjusted odds ratio from logistic mixed effects model adjusted for baseline outcome value = 1.33[0.78–2.26] (ns)
						Employment status at 6 months	Higher proportion of the intervention group unemployed (77.9% vs 72.9%). Adjusted odds ratio from logistic mixed effects model adjusted for baseline outcome value = 1.41 [0.82–2.42] (ns)
Hall et al. (2017)	Recovering from addiction 100% men 51% African American 100% prison history Mean age 43.6	Financial incentive Community based Employment included within treatment/support services Monetary vouchers for attending 5-month residential programme with therapeutic community model. Services to address various needs, with an employment focus. Usually group sessions, participants learn to follow rules; work on substance use, mental health, family issues; attended AA/NA meetings; address criminal thinking; have access to education and work activities, learn skills and attitudes that will help “beat the streets”	Usual services	202	18	Any employment 6 months prior to follow up	Both groups had employment rates of 31% (ns)
Jason et al. (2015)	Recovering from addiction Excluded people with violent or sexual offences 83% men 74% Black/African American 100% prison history Mean age 38.8–43.3	a) Oxford Houses (OH) b) Therapeutic community (TC) Community based Employment included within treatment/support services Oxford Houses are residential facilities led by peers, requiring abstinence from alcohol/drugs, rent payment and weekly chores Therapeutic community is professionally led abstinence orientated residential treatment service where participants follow a structured substance use recovery plan including self-help groups, calling a sponsor and random drug screening tests	Usual services	270	24	Days worked in last month at 6, 12, 18 & 24 months	General linear mixed model found significant effects for condition (F = 3.60, df = 2937, *p* < 0.03), time (F = 83.44, df = 1937, *p* < 0.01), and time by condition interaction (F = 4.41, df = 2937, p = 0.01)
LePage et al. (2016)	Mental health and/ or substance use disorder (excluding active psychosis) Veteran 96% men 68% African American 100% prison history Mean age 52.3	Modified IPS + About Face Community based Employment focused IPS as above Modified to require participation in pre-employment classes (About Face), allow employment specialists to carry larger caseloads, and no integration with a mental health treatment team	About Face	111	6	Achieved any employment at 3 months	33% intervention vs 16% control (ns)
						Achieved any employment at 6 months	46% intervention vs 21% control (x^2^ = 5.9, df = 1, OR = 3.5, *p* < 0.05). Significance maintained when controlling for time since last full-time employment (x^2^ = 4.1, OR = 2.9, *p* < 0.05)
						No days employed	43.8(58.0) intervention vs 20.7(45.6) control. (MWU, p = 0.03)
						Hours worked/week	17.4(25.6) intervention vs 7.9(17.4) control (MWU, *p* = 0.04)
						Total hours worked	130.1(222.7) intervention vs 52.3(130.6) control (MWU, *p* = 0.3)
LePage et al. (2020)	Mental health and/ or substance use disorder (excluding active psychosis) Veteran 96% men 76% racial/ethnic minority Mean age 52	Modified IPS + About Face Vocational Program Community based Employment focused IPS as above Modified to require participation in pre-employment classes (About Face Vocational Program), allow employment specialists to carry larger caseloads, a focus on ‘conviction friendly’ professions, and no integration with a mental health treatment team	About Face Vocational Program	88	12	Achieved any employment	57% intervention vs 37% control (OR = 2.20, 95% CI = 1.03–4.7, p = 0.046)
						Stable employment	42% intervention vs 29% control
						Fulltime employment (35 + hrs./week)	43% intervention vs 18% control (OR = 3.56, *p* = 0.006)
						No months employed	4.2(3.8) intervention versus 2.3(4.6) control (t = 2.2, df = 109, *p* = 0.027)
						No hours worked	281.2(476.9) intervention vs 569.1(668.3) control (MWU = 1888, *p* = 0.03)
Polcin et al. (2018)	HIV + status Recovering from addiction 74% men 47% white 100% prison history Mean age 38.6	Motivational Interviewing Case Management (MICM) in Sober Living Houses Community based Employment included within treatment/support services Motivational interviewing techniques applied to support case management, aiming to help people anticipate and respond to challenges as they transition into a new living situation, seek employment, and access community services	Sober Living Houses as usual	330	12	Addiction severity index employment scale 12 months (paper also reports 6 months)	0.76(0.26) intervention vs 0.73(0.27) control
						Days worked last 6 months at 12 months (paper also reports 6 months)	44.97(57.82) intervention vs 49.08(60.05) control (ns in multilevel model)
Smith et al. (2022)	General population 100% men 46–56% Black/African American 100% prison history Mean age 38.1–39	Virtual Reality Job Interview Training (VRJIT) and prison ‘vocational village’ Prison based Employment focused E-learning curriculum that introduces eight job interview skills and computerized job interviews across three levels of difficulty with the interviewer displaying different personalities (friendly, professional, inappropriate)	‘Vocational village’ as usual	44	6	Achieved any employment	Intervention group had significantly better odds after covarying for age, race, time served in prison/jail, arrests, prior violent crime, psychological distress, and pre-test interview skill. (OR = 7.4, 95% CI = 1.1–51.4, *p* = .045)
						Hours worked/week	39.6(2.1) intervention vs. 41.0(3.2) control (t = 1.0, *p* = 0.32)
Webster et al. (2014)	Recovering from addiction/using drugs 65% men 61% white 80% prison history Mean age 29.7–31.2	Employment intervention + drug court Community based Employment focused An employment intervention delivered as 26 group and individual session wtih focus on employment barriers and resolving issues that impede employment success over 3 phases: obtaining, maintaining, and upgrading employment. Includes behavioural contracting, motivational interviewing, and strengths-based case management	Drug court as usual	500	12	Ave. employment status last 12 months	Comparing between 4 categories (*p* = 0.056) (ns) Full or part time on average 80.5% intervention vs 75.6% control (x^2^[3, *N* = 447] = 9.67, *p* = 0.022, ϕ = 0.15)
						Days employed last 12 months	210.1(114.1) intervention vs 199.9 (130.1) control (d = 0.20 F[1, 464] = 4.69, *p* = 0.03)
						Days employed last 30 days	17.8 intervention vs 16.1 control (sd and statistical tests not reported)

*Abbreviations*: *AOR* Adjusted odds ratio, *b* beta coefficient, *CI* Confidence intervals, *d* effect size, *df* degrees of freedom, *F* f test statistic, *MWU* Mann Whitney U test, *ns* not significant, *OR* Odds ratio, *p* probability, *sd* standard deviation, *t* t test statistic, *x*^*2*^ chi squared test statistic

#### Design

The included studies were: nine two-arm parallel RCTs (Bond et al., 2015; Cook et al., 2015; Duwe, 2012; Farabee et al., 2014; Fogel et al., 2015; Hall et al., 2017; LePage et al., 2016, 2020; Webster et al., 2014); one three-arm parallel RCT (Jason et al., 2015a); and one cluster RCT (Polcin et al., 2018). One study was described as a ‘feasibility study and initial effectiveness trial’ (Smith et al., 2022). Participant numbers ranged from 44–521.

#### Settings

All studies were conducted in the USA. Two interventions were conducted in prison (Fogel et al., 2015; Smith et al., 2022), two spanned both prison and community (termed transitional) (Cook et al., 2015; Duwe, 2012), and the remaining eight were community-based. Three community-based interventions were within residential treatment services for addiction recovery, with varying levels of peer involvement (Hall et al., 2017; Jason et al., 2015a; Polcin et al., 2018).

#### Population

Three studies were men only (Cook et al., 2015; Hall et al., 2017; Polcin et al., 2018). Eight studies were majority (65–96%) men (Bond et al., 2015; Duwe, 2012; Farabee et al., 2014; Jason et al., 2015a; LePage et al., 2020; LePage et al., 2016; Polcin et al., 2018; Webster et al., 2014). One study included women only (Fogel et al., 2015). Studies all had ethnically diverse participants, with half (*n* = 6) having a clear majority of participants from a racial/ethnic minority (Bond et al., 2015; Cook et al., 2015; Duwe, 2012; Jason et al., 2015b; LePage et al., 2020; LePage et al., 2016), and the remainder having variation between different ethnic groups. Only Bond et al. (2015) and Webster et al. (2014) had participants who were not formerly in prison, representing 24% and 20% of their samples respectively.

Seven studies focused on specific sub-populations of people who had been in prison: people with severe mental illness (Bond et al., 2015), veterans with mental illness and/or substance use disorder (LePage et al., 2016, 2020), people currently using substances or on abstinence programs (Hall et al., 2017; Jason et al., 2015a; Webster et al., 2014), and people with HIV + status living in abstinence based facilities (Polcin et al., 2018). Five studies recruited a sample of the general prison population (Cook et al., 2015; Duwe, 2012; Farabee et al., 2014; Fogel et al., 2015; Smith et al., 2022).

#### Interventions

Three studies tested Individual Placement and Support (IPS) (Bond et al., 2015; LePage et al., 2016, 2020). The two studies by LePage and colleagues involved a modified IPS (mIPS). The remaining nine studies each tested a different intervention (see Table 1 for results of each and a summary of content).

Six interventions focused on employment only (Bond et al., 2015; Farabee et al., 2014; LePage et al., 2016, 2020; Smith et al., 2022; Webster et al., 2014), five included employment but with wider support (Cook et al., 2015; Duwe, 2012; Hall et al., 2017; Jason et al., 2015a; Polcin et al., 2018) and one had no employment component (Fogel et al., 2015).

#### Comparators

In the studies focused on a specific population, three were receiving a specialist service as’treatment as usual’ (Hall et al., 2017; Polcin et al., 2018; Webster et al., 2014). Three studies compared IPS or mIPS to a comparator vocational intervention within a specialist service (Bond et al., 2015; LePage et al., 2016, 2020). In the mIPS studies, those receiving mIPS also attended the control vocational intervention before commencing mIPS. Jason et al. (2015) had two trial arms receiving an intervention (sober living facility or therapeutic community) and one trial arm that received no additional services.

In studies with the general population, comparators were no other intervention (Cook et al., 2015; Duwe, 2012), a list of resources and a meal voucher (Farabee et al., 2014), a single sexually transmitted infection prevention session (Fogel et al., 2015) and in one case the intervention and control group were receiving enhanced vocational services within prison, accessible to people with high behavioural standards (e.g., six months free of serious prison incidents) and who had achieved set academic milestones (Smith et al., 2022).

### Outcomes

Thirty-six employment outcomes were reported across the 12 studies. These included whether someone achieved any employment (even for one day), the amount of time worked over a set period (measured in different units), average employment status over different periods of time, employment status at a point in time, number of people achieving ‘stable competitive employment’ and employment scores on an assessment scale. Several studies had multiple ways of measuring employment. None reported the type of work people obtained, such as by industry or skill level. Outcomes per study are reported in Table 1.

### Risk of bias in studies

Seventeen of the outcomes were judged to be of high risk of bias. These were from studies where randomization was insufficiently described (Duwe, 2012), per protocol analysis (only participants who complete the treatment are included in final comparisons, thus introducing bias by omitting those who dropped out and had potentially less favourable outcomes) was conducted (LePage et al., 2016, 2020), or there were challenges delivering the intervention as intended (Polcin et al., 2018). Results for sixteen outcomes were rated as ‘some concerns’ related to bias, and results for three outcomes were rated low risk of bias. Most studies did not have pre-published or available protocols (resulting in a ‘some concerns rating’) which would have allowed appraisal of any bias introduced by post hoc selection of outcome measures or changes in method. See Supplementary File 2 for the full ROB2 results.

### Results of individual studies

Studies often reported more than one outcome at several timepoints. Eight studies reported a significant effect in favour of the intervention on at least one employment outcome at the endpoint of the study (Bond et al., 2015; Cook et al., 2015; Duwe, 2012; Jason et al., 2015a; LePage et al., 2020; LePage et al., 2016; Smith et al., 2022; Webster et al., 2014). Four reported no significant results (Farabee et al., 2014; Fogel et al., 2015; Hall et al., 2017; Polcin et al., 2018). Individual study results are summarized in Table 1.

### Results of syntheses

**Fig. 2:**
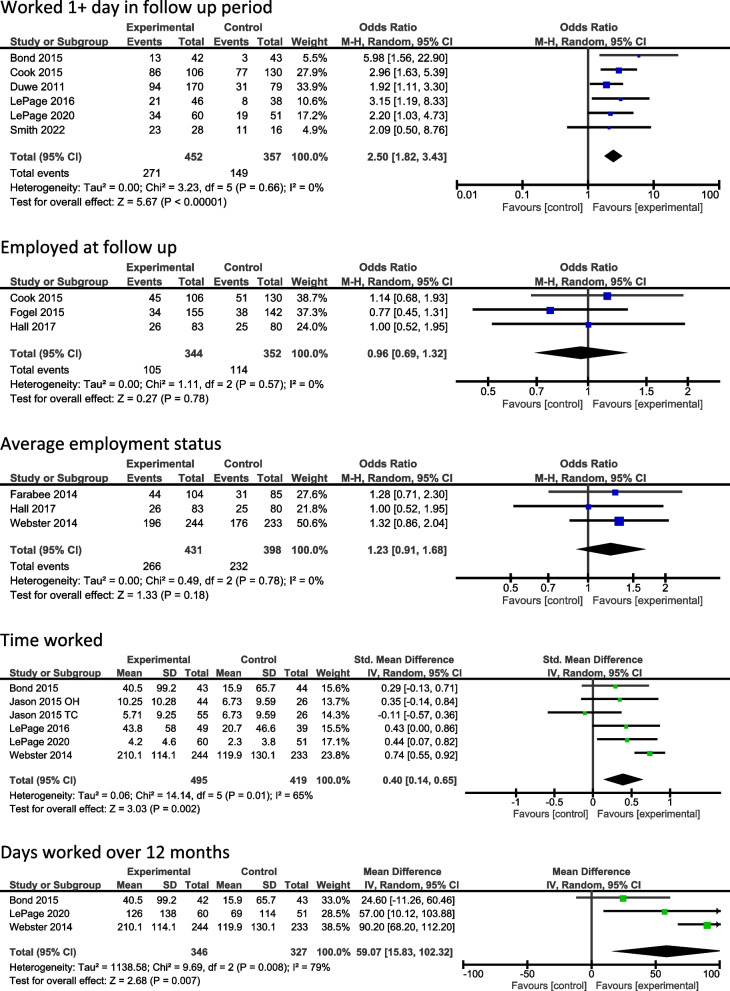
Forest Plots

We identified four employment outcomes which were reported across three or more studies: 1) number of participants who worked any time in the follow up period, 2) number of participants in employment at the follow up point, 3) whether participants were typically in employment throughout the follow up period, and 4) amount of time worked. Forest plots for each comparison are included in Fig. 2. Confidence intervals (CI) for all comparisons were 95%.

#### Achieved any employment in follow up period

Six studies reported if participants achieved employment for at least one day during the follow up. Three followed up participants for six months (Duwe, 2012; LePage et al., 2016; Smith et al., 2022) and three for 12 months (Bond et al., 2015; Cook et al., 2015; LePage et al., 2020).

Overall, those receiving an employment intervention were 2.5 times more likely to work one or more days than those in the control group (CI 1.82–3.43). Odds ratios (OR) were slightly higher in studies that measured outcomes at 12 months (6 months OR = 2.15, CI = 1.37–3.37 vs 12 months OR = 2.89, CI = 1.85–4.51). Heterogeneity was low (I^2^ = 0%). Excluding Smith et al. (2022) from comparisons due to small sample size and Covid-19 related disruption to the study did not substantially change these estimates (OR = 2.52, CI = 1.82–3.49).

In subgroup analysis focusing on those interventions delivered in the community only (which also served as a comparison of all the studies of IPS/mIPS), overall odds and heterogeneity were increased to 2.91 (CI = 1.68–5.034, I^2^ = 40%). However, the two mIPS studies were conducted per protocol and this result must be considered cautiously. In studies that recruited a general population (rather than a specific subgroup of people), which made up the remaining three interventions (two transitional and one prison only) odds were 2.31 (CI = 1.57–3.41, I^2^ = 0%). Examining transitional interventions only (albeit there were only two), odds were 2.34 [CI = 1.53–3.57, I^2^ = 10%].

Excluding studies of high risk of bias increased odds to 3.14 (CI = 1.88–5.23, I^2^ = 0%). This was largely due to exclusion of one large study (Duwe, 2012). Duwe (2012) was not explicit about how outcome data were reported. Excluding this study to leave only those with validated outcome data increased the odds ratio (OR = 2.86, CI = 1.94–4.22).

Based on the GRADE assessment, we determined there to be moderate certainty that employment interventions can increase the number of people who achieve employment for at least one day.

#### Employed at follow up point

Three studies reported the employment status of participants at the final follow up point. Cook et al. (2015) reported employment status at 12 months in a transitional intervention, Fogel et al. (2015) at six months in a prison-based intervention and Hall et al. (2017) measured any employment in the six months prior to data collection at 18 months in a community-based intervention. The latter is included here rather than in ‘achieved any employment in follow up period’ as there are no data for the preceding 12 months.

There was no evidence of an effect on employment at follow up (OR = 0.96, CI = 0.69–1.32). Heterogeneity was low with I^2^ = 0%. Subgroup analyses were conducted based on those studies recruiting a general prison population as opposed to a specific subgroup (Cook et al., 2015; Fogel et al, 2015) although this was based on two studies only. It did not change the overall result (OR = 0.94, CI = 0.64–1.39). Two studies had self-report outcome data. None were high risk of bias. We assessed the certainty of this result as moderate due to the small number of studies and slight difference in outcome measurement.

#### Average employment status

Three studies compared average employment status. Farabee et al. (2014) and Webster et al. (2014) consider the last 12 months and Hall et al. (2017) the last 6 months. All were community-based interventions.

No evidence of effect was identified (OR = 1.23, CI = 0.91–1.68). Heterogeneity was low with I^2^ = 0%. Subgroup analyses were conducted on studies with people with substance use needs (Hall et al., 2015; Webster et al., 2014), but this was based on two studies only and did not change the overall result (OR = 1.22, CI = 0.85–1.75). All studies relied on self-report employment data. None were high risk of bias. We graded our certainty in this result as moderate due to the small number of studies and different follow up periods.

#### Time worked

Seven comparisons from six studies reported data pertaining to time worked measured in hours, days, weeks or months. Jason et al. (2015a) reported the last month, two studies reported time worked over six months (LePage et al., 2016; Smith et al., 2022), and four reported over 12 months (Bond et al., 2015; LePage et al., 2020; Polcin et al., 2018; Webster et al., 2014). Smith et al. (2022) was the only prison-based intervention in the comparison.

Polcin et al. (2018), as a cluster RCT, was not included in the meta-analysis of this outcome. They observed no significant effect on the number of days worked in the last 6 months, measured at six and 12 months when running time by condition comparisons in a multi-level model controlling for demographic variables (exact results not reported). Data presented indicates that those in the intervention group worked fewer days than those in the control condition at all time points, and that both groups had a significant increase in number of days worked.

Overall, heterogeneity was high, and we did not find a clear effect in favour of the intervention (SMD = 0.2, CI = -0.13–0.52, I^2^ = 86%). This was primarily due to one study conducted in prison only, with a small sample size and which was disrupted by Covid-19 (Smith et al., 2022). Excluding this study showed a clear effect in favour of the interventions with heterogeneity reduced to moderate (SMD = 0.40 CI = 0.14–0.65, I^2^ = 65%).

Subgroup analysis of studies testing IPS/mIPS against a comparator vocational intervention showed an effect in favour of IPS/mIPS (SMD = 0.39, CI = 0.16–0.63, I^2^ = 0%). Two of three studies included used per protocol analysis and were considered high risk of bias. When excluding Smith et al. (2022) and studies that relied on self-report employment data, there was a clear effect in favour of the intervention (SMD = 0.39 CI = 0.16, 0.63, I^2^ = 0). However, this comparison included studies of high risk of bias. Continuing to exclude Smith et al. (2022) whilst excluding studies with high risk of bias reduced the effect to a non-significant level and increased heterogeneity to high (SMD = 0.35 CI = -0.05, 0.75, I^2^ = 79%). However, this drew from several studies with self-report data.

Certainty in the result was graded as low due to the inclusion of studies with a high risk of bias and self-report of outcome data.

Three studies reported the time worked over twelve months (Bond et al., 2015; LePage et al, 2020; Webster et al., 2014). We converted data from months to days for one study (LePage et al., 2020). This comparison also showed a clear effect in favour of intervention with moderate-high heterogeneity (MD = 59.07 days, CI = 15.83–102.32, I^2^ = 79%). This was sustained when removing a high risk of bias study, and again when removing the study using self-report outcome data. Certainty in this estimate was rated as moderate.

## Discussion

We identified 12 RCTs, all conducted in the USA, that reported employment outcomes for people released from prison. These studies used a range of measures at different time points, with many studies using multiple outcomes and none reporting the type or quality of employment obtained. Meta-analyses indicated employment interventions are effective for increasing the number of people who achieve employment in the follow up period. We have moderate certainty in the estimate. We found a clear effect in favour of interventions for increasing the amount of time worked when excluding one study with limitations. We had low confidence in this estimate. However, we had moderate confidence that interventions can increase the number of days worked over a 12-month intervention period. No clear effect was seen for average employment status, or employment status at the end point. We have moderate confidence in both estimates.

### General Interpretation in context of other evidence

Interventions were effective for supporting people to achieve employment during the follow up period and increasing the time worked over the follow up period. However, they were not effective for increasing the proportion of participants in work at the end point or average employment status. The latter two require at least some *sustained* employment, indicating that interventions may be supporting people to find and start work, but not to maintain it. This finding has been replicated in studies of employment interventions for people with mental illnesses (Barnett et al., 2022). Reasons for not sustaining employment could be that the jobs themselves are less stable (zero-hours and fixed term opportunities) or barriers and stigma within the workplace (Sheppard & Ricciardelli, 2020). However, the type of jobs people obtained were not reported in the included papers. Others suggest that people released from prison may not be adequately prepared with appropriate ‘soft’ and ‘life’ skills to achieve and sustain work (Bain, 2019). Alternatively, the stresses of adapting to life post-imprisonment more broadly may impact on the feasibility of sustaining employment. People released often return to marginalized communities with few resources (Morenoff & Harding, 2014). When experiencing multiple difficulties over the adaptation period, with limited social and community support, sustaining employment may be deprioritized with potential consequences for health and reoffending.

It is positive that employment interventions are enabling people to access employment more readily, however the health and financial benefits to the person and society may be lost unless interventions also address job maintenance and quality. Ensuring the jobs people access are stable and good quality (less interchangeability of staff, higher income) is also important for achieving any protective effect against reoffending (Ramakers et al., 2017). Neglecting this may contribute to the equivocal finding about the effectiveness of employment interventions for reducing recidivism (Visher et al., 2005). In the absence of evidence-based interventions that support people to achieve *stable* employment, practitioners may consider targeting workplace environments and stigma by working with employers, encouraging people to apply for good quality stable employment, addressing ‘soft’ and ‘life skills’ that would support sustaining a job, and supporting wider needs related to health and residing in under-served communities. However, as we demonstrated, there is a need for significant research in this space to convert potential intervention targets into rigorous interventions that can be evaluated to determine their effectiveness, and therefore inform practitioners’ efforts to support people who have been in prison into employment.

We identified three studies testing IPS or a modified version of it. A meta-analysis of IPS among people with mental illnesses estimated a pooled risk ratio for finding any employment of 2.40 (Modini et al., 2016). The comparable risk ratio from the IPS/mIPS studies in our review was 1.44, suggesting that IPS may need more specific modifications to achieve the same effects when someone living with mental illness also has an imprisonment history, for example in negotiating disclosure of a criminal record and supporting adaptation to post-imprisonment life. However, our estimate is based on two studies of high risk of bias in a way that may overestimate effectiveness. Further research is required into IPS with people released from prison to draw clearer conclusions.

Finally, in addition to intervention variation, there was substantial variation between studies in our review in how employment was operationalised and the time point at which outcomes were measured. Study design moderates the effect size identified when measuring recidivism following prison-based interventions (Nur & Nguyen, 2022) and thus may also impact the effects observed in employment outcomes. Our inclusion of community-based studies and more nuanced evaluation of how study design, setting and outcome operationalisation influence effect sizes presents a valuable addition to understanding intervention effects on employment outcomes. However, there is a need for a greater number of studies using randomised designs to replicate and further refine these understandings. For example, utilising the same outcome measures at the same time point and concentrating on stable and good quality employment.

### Limitations of evidence included

Almost all the outcomes were rated as having some concerns in relation to bias, with 17/36 rated as a high risk of bias. However, we ran sensitivity analyses excluding these. Employment levels at baseline (including recent employment) were not always clear, and we thus could not fully appraise the comparability of the groups (e.g., if they included people working part time, or people who had recent employment histories). We thus assumed that participants were comparable on this basis. It was not clear in some studies how recently someone had been released from prison. The likelihood of intervention success may differ between the immediate post-release period compared to when someone has achieved some level of adaptation to community life before engaging with an intervention. Finally, we were not able to identify the type or quality of employment people achieved and the relevance of this for sustainability.

All the studies were conducted in the USA. Thus, caution is required when considering the transferability of the effect estimates and interventions to contexts with substantially different justice systems, legislative and policy landscapes, labor markets, social and economic conditions and social support systems. Only one study involved women only, with the remainder having none or few women participants. There may be different considerations for men and women that cannot be established from these studies.

### Limitation of review processes

We conducted our search in English, which may have resulted in the omission of studies if an English language abstract was not available. However, the contemporary practice of making abstracts available in English (and our translation of studies identified this way) indicates this limitation is moderated to an extent. We did not include grey literature as we intended to include only high-quality peer reviewed RCTs conducted to international standards, which are typically reported in journals. This may have overlooked RCTs reported only in the grey literature; however, this is unlikely given contemporary reporting standards and our focus on publications since 2010.

We calculated odds ratios, which can overestimate results compared to risk ratios when an outcome is relatively common (Davies et al., 1998). Running our analysis to calculate risk ratios did not change the overall significance of our results or the qualitative conclusions drawn from them. Despite low levels of heterogeneity according to I^2^ in several comparisons, most of these included a small number of studies and thus uncertainty around the I^2^ estimates should be considered substantial (Deeks et al., 2019; von Hippel, 2015). Nonetheless, given the heterogeneity between studies we ran random effects models and conducted subgroup analyses exploring potential sources of heterogeneity.

Several of the studies of employment outcomes utilised administrative data or validated self-reports of employment, increasing our confidence in their accuracy. We conducted a sensitivity analysis to exclude studies that used self-report data only, to address the risk that this may be biased by participant attempts to manage researchers’ impressions of their success, and found no substantial differences in our results.

### Implications for practice, policy, and research

We have moderate certainty that interventions can increase the number of people who start employment, and the number of days worked over a 12-month period. Given the health benefits and protection against reoffending, service providers should make employment interventions available to people released from prison. However, it is crucial that these interventions focus not just on starting a job, but on ensuring that work is of good quality and that the individual has the skills and support to sustain employment. Barriers described in the literature may form useful intervention targets (collaborating with employers to modify workplace environments and reduce stigma, encouraging people toward good quality stable employment, and simultaneously addressing ‘life skills’ and wider needs such as poor health). However, there is currently a lack of clarity about whether strategies such as these are likely to be effective. Research is urgently needed to develop and rigorously evaluate interventions for their effects on sustained good quality employment. Policymakers within and outside the USA are limited in the evidence available to them with regards to effective strategies for supporting people into employment, and thus may consider commissioning rigorous independent research evaluation of existing employment programmes to ensure optimal policy decisions.

To enhance the ability to commission and provide effective interventions, research should establish not only effective interventions, but also what intervention components are necessary for interventions to be effective for achieving stable, good quality employment. Research is particularly needed outside the USA and it would be beneficial to consider the needs of different populations within the justice system, including women. Future RCTs should use consistent measures across studies at the same time point. These should be outcomes considered a meaningful indicator of employment by people receiving interventions (e.g., satisfying/stable work). We suggest that employment rate measured at regular intervals would give a more meaningful indication of an interventions effect on stable employment, and consideration should be given to developing indicators of good quality jobs.

## Conclusion

Interventions are effective for increasing the number of people who start employment and the amount of time worked following release from prison, and therefore should be made available given the benefits of employment to health and protection against reoffending. No evidence of effectiveness was found on indicators of sustained employment. Interventions provided must focus on securing stable, good quality work that a person has the skills to sustain, however evidence of what such interventions should consist of to be effective is limited at present. Confidence in making recommendations about interventions to improve employment could be increased with high quality RCTs conducted outside the USA that measure employment rates over time- and clearly describe the components.

## Supplementary Information

**Additional file 1.**

**Additional file 2.**

**Additional file 3. **

**Additional file 4.**

## Data Availability

Materials used in the review (template data collection forms; data extracted from included studies; data used for analyses) are available from the corresponding author upon reasonable request.
