# Supplementary material for: Effectiveness of interventions to improve employment for people released from prison: systematic review and meta-analysis
Source: Health Justice. 2023 Mar 14;11:17. doi: 10.1186/s40352-023-00217-w (PMC10010959; doi:10.1186/s40352-023-00217-w)
Supplement: Supplementary file 1 — Additional file 1. [file 40352_2023_217_MOESM1_ESM.docx]

# Search strategy

All searches used the following strategy adapted to database requirements. Key words were mapped to database specific subject headings, and searched as text

The full search from EMBASE is shown below

## Template – key terms/key words

|  |  | **Key words** |  |
| --- | --- | --- | --- |
| 1 | Population: Justice involved adults | Offender  Criminal  Delinquent  Gang  Felon | Perpetrator  Ex-prisoner  Probationer  Parolee |
|  |  | offen* OR crim* OR delinq* OR felon* OR gang OR perpetrat* OR justice* or prison* or probation* or parole* | |
| 2 | Study type: Randomised Controlled Trials | Randomised  Blind  Controlled | Trial  Experiment |
|  |  | random* or “randomised controlled trial” or “RCT” or blind* or “controlled trial” or experiment* | |
| S3 | Outcome: Social outcome (participation in activity or social role) | Time use  Activities  Occupation  Self-care  Work / Employment  Volunteering  Vocational training  Education  Leisure  Recreation  Sport | Hobbies  Faith  Religion  Spirituality  Social functioning  Social participation  Civic participation  Citizenship  Parent/Mother/Father  Friend  Interpersonal relationship |
|  |  | "time use" or “time-use” or activit* OR occupation* OR self-care OR work* OR employ* OR volunteer* OR vocation* OR education* OR leisure OR recreat* OR sport* OR hobb* OR faith OR religio* OR spiritual* OR "social function" or “social functioning” OR “social participation” or “civic participation” or citizenship or parent* or mother* or father* or friend* or “interpersonal relationship” or “interpersonal relationships” | |
| 4 | Setting: Community | Probation  Parole  Release  Discharge  Integration/reintegration | Rehabilitation  Desistance  Re-entry  Re-settlement  Post release |
|  |  | Probation* or parole* or release* or discharge* or integrat* OR reintegrat* OR rehabilitat* OR desist* OR reent* OR re-ent* OR re-settl* OR resettle* or postrelease or “post-release” | |
| 5 | Population: exclude under 18s | Youth  Adolescent  Teenager | Child  Juvenile |
|  |  | NOT (youth or adolescen* or teen* or child* or juvenile*) | |
| 6 | 1 AND 2 AND 3 AND 4 |  |  |
| 7 | 6 NOT 5 |  |  |
| 8 | Date limiters | Dates 2010 onwards |  |

## Embase example


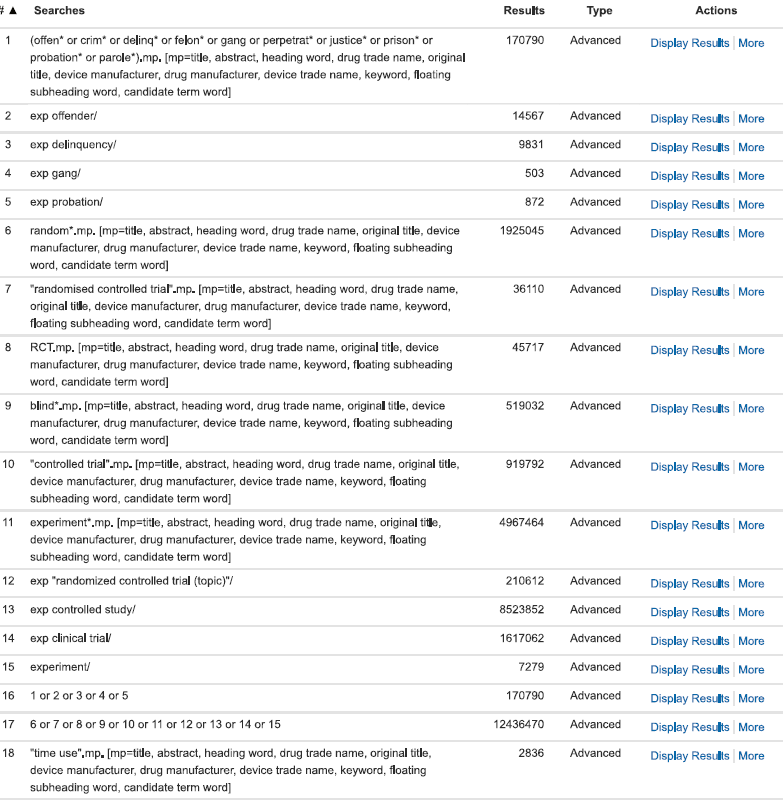


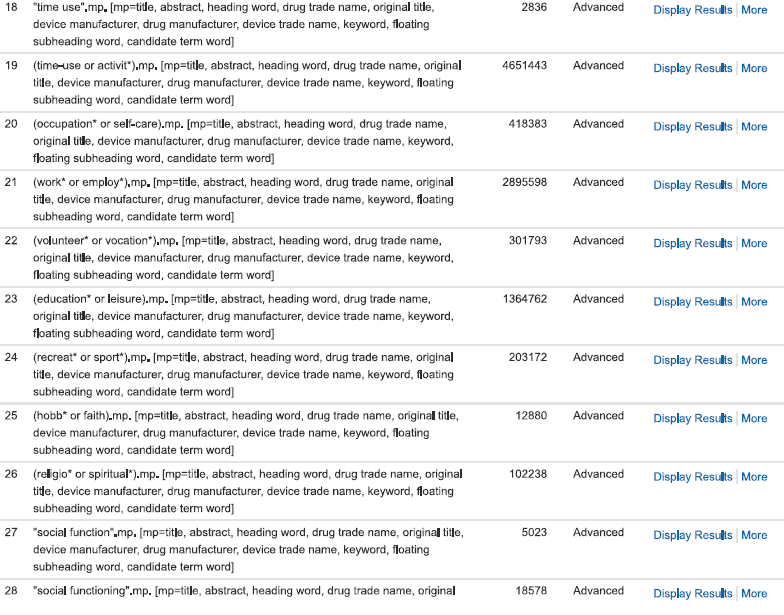


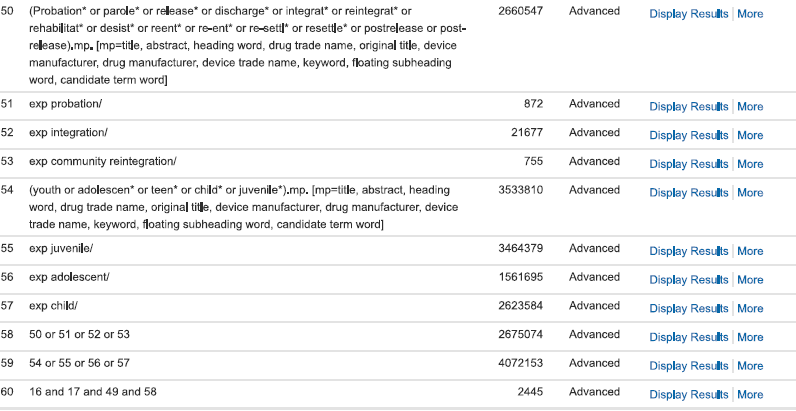

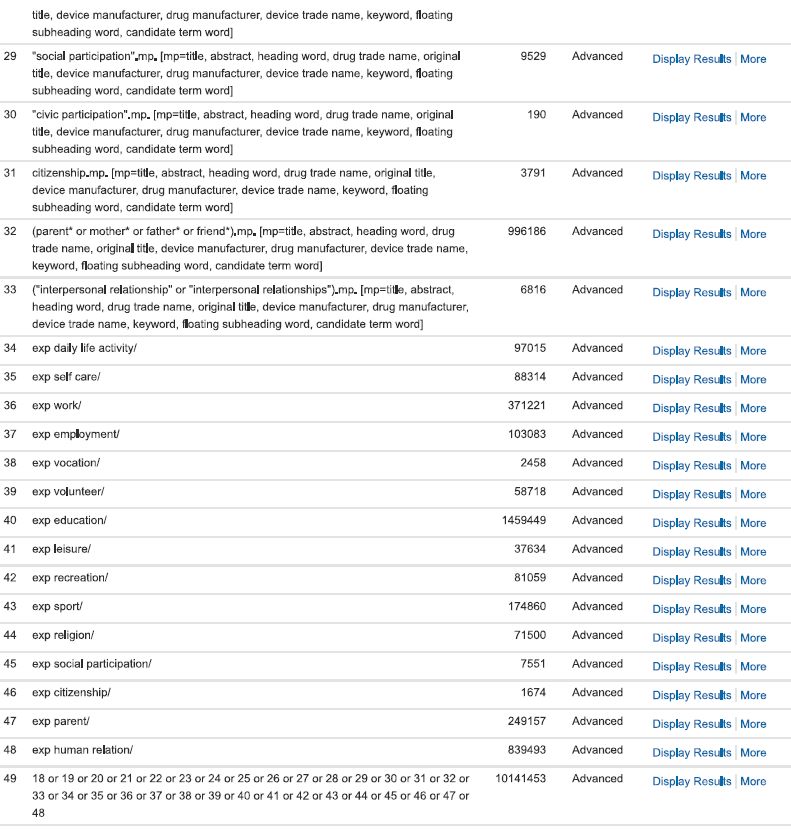


*From 60 – we applied limits of dates and selected adults in to get final result
