# Supplementary figures and images for: Effectiveness of interventions to improve employment for people released from prison: systematic review and meta-analysis
Source: Health Justice. 2023 Mar 14;11:17. doi: 10.1186/s40352-023-00217-w (PMC10010959; doi:10.1186/s40352-023-00217-w)

## Risk of bias assessment


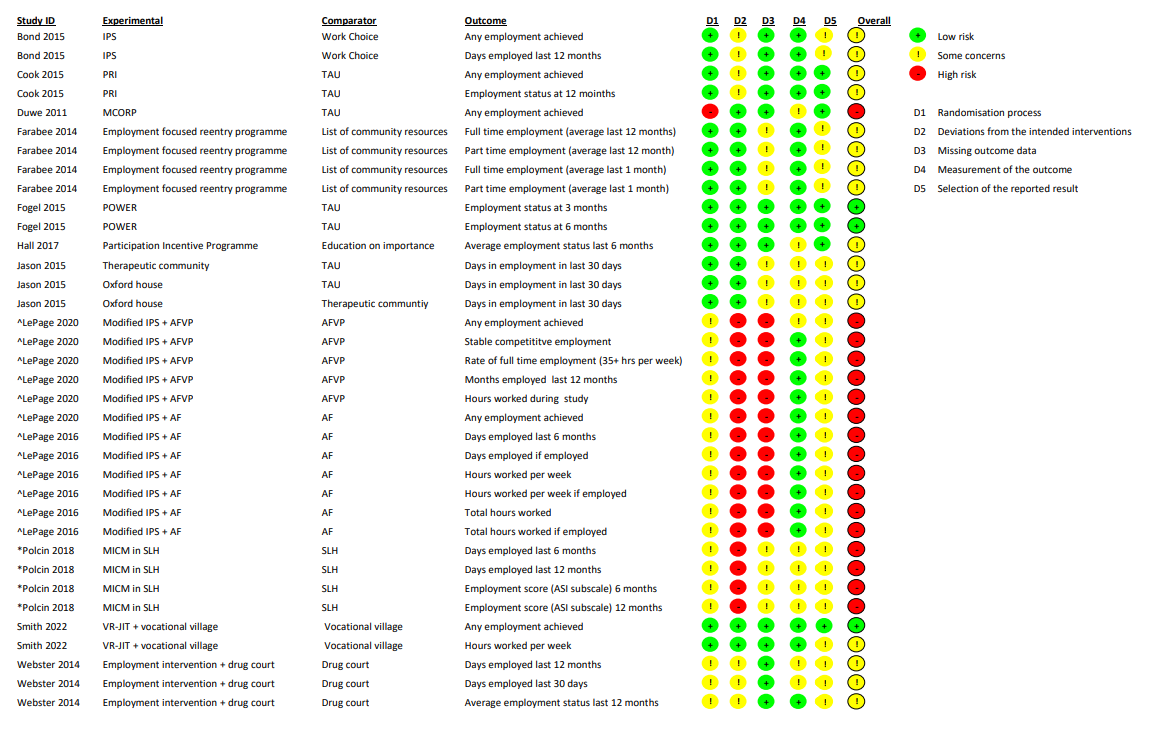


*=cluster RCT

^=per protocol analysis

Supplement: Supplementary file 2 — Additional file 2. [file 40352_2023_217_MOESM2_ESM.docx]
