# Supplementary material for: Effectiveness of interventions to improve employment for people released from prison: systematic review and meta-analysis
Source: Health Justice. 2023 Mar 14;11:17. doi: 10.1186/s40352-023-00217-w (PMC10010959; doi:10.1186/s40352-023-00217-w)
Supplement: Supplementary file 3 — Additional file 3. [file 40352_2023_217_MOESM3_ESM.docx]

# GRADE table

Question: Should employment interventions be provided for people released from prison

| Certainty assessment | | | | | | | № of patients | | Effect | | Certainty | Importance |
| --- | --- | --- | --- | --- | --- | --- | --- | --- | --- | --- | --- | --- |
| № of studies | Study design | Risk of bias | Inconsistency | Indirectness | Imprecision | Other considerations | employment interventions | usual after care | Relative (95% CI) | Absolute (95% CI) |  |  |
| **Worked any time in the follow up period** | | | | | | | | | | | | |
| 6 | randomised trials | serious^a^ | not serious^b^ | not serious^c^ | not serious^d,e^ | none | 271/452 (60.0%) | 149/357 (41.7%) | **OR 2.50** (1.82 to 3.43) | 224 more per 1,000 (from 149 more to 293 more) | ⨁⨁⨁◯ Moderate | IMPORTANT |
| **Employed at follow up point** | | | | | | | | | | | | |
| 3 | randomised trials | not serious | not serious^b^ | not serious^c^ | serious^e,f^ | none | 105/344 (30.5%) | 114/352 (32.4%) | **OR 0.96** (0.69 to 1.32) | 9 fewer per 1,000 (from 75 fewer to 63 more) | ⨁⨁⨁◯ Moderate | IMPORTANT |
| **Typically in employment** | | | | | | | | | | | | |
| 3 | randomised trials | not serious | not serious^b^ | not serious^c^ | serious^g^ | none | 266/431 (61.7%) | 232/398 (58.3%) | **OR 1.23** (0.91 to 1.68) | 49 more per 1,000 (from 23 fewer to 118 more) | ⨁⨁⨁◯ Moderate | IMPORTANT |
| **Time worked** | | | | | | | | | | | | |
| 6 | randomised trials | serious^a^ | not serious^h^ | not serious^c^ | serious^e^ | none | 495 | 419 | - | SMD **0.4 higher** (0.14 higher to 0.65 higher) | ⨁⨁◯◯ Low | IMPORTANT |
| **Days worked in last 12 months** | | | | | | | | | | | | |
| 3 | randomised trials | serious^a^ | not serious^i^ | not serious^c^ | not serious^j^ | none | 346 | 327 | - | MD **59.07 higher** (15.83 higher to 102.32 higher) | ⨁⨁⨁◯ Moderate |  |

**CI:** confidence interval; **MD:** mean difference; **OR:** odds ratio; **SMD:** standardised mean difference

a. Includes studies with high risk of bias

b. Point estimates are not widely spread and confidence intervals overlap for all studies. Low-moderate heterogeneity

c. There were differences in intervention location (community or prison) and population. However all were employment interventions conducted in the USA for people with a history of imprisonment.

d. Confidence intervals of the overall effect are not wide and fall clearly in favour of the intervention

e. Follow up period differed between studies

f. Confidence intervals are not wide, but cross the midline between favouring intervention or favouring control.

g. Wide confidence intervals around effect, which cross the midline between favouring intervention and favouring control.

h. Majority of studies have overlapping confidence intervals and relatively close point estimates. Heterogeneity is explained by differences in intervention and population

i. Point estimates all in favor but two not overlapping. Moderate to high heterogeneity: Tau² = 1138.58; Chi² = 9.69, df = 2 (P = 0.008); I² = 79%

j. Wide effect estimate but clear in direction
